# Embracing Complexity Within Creative Approaches to Dementia Research: Ethics, Reflexivity, and Research Practices

**DOI:** 10.1177/16094069231165932

**Published:** 2023-04-02

**Authors:** Sarah Campbell, Robyn Dowlen, Rebecka Fleetwood-Smith

**Affiliations:** 1 Social Care and Social Work, Faculty of Health and Education, Manchester Metropolitan University, Manchester, UK; 2 Division of Nursing, Midwifery and Social Work, University of Manchester, Manchester, UK; 3 Department of History, University of Bristol, Bristol, UK

**Keywords:** creative research methods, creative research approaches, dementia, reflexivity, ethics, attunement, sensory, embodiment, complexity

## Abstract

In this paper, we present reflections from three research studies that have engaged with creative approaches to qualitative research with people living with dementia. Creative approaches to qualitative research are increasingly advocated within dementia research as they foreground alternative routes to expression and can facilitate flexible, meaningful participation. Such approaches are typically cited as illuminating people’s lived experiences as they often enable nuanced understandings around how people with dementia engage in the world around them. Yet creative approaches to research with people living with dementia involve specific complexities that require rigorous planning, generous timelines, and interdisciplinary research teams. It is important to examine the role of the researcher(s) in the development and application of creative approaches, as this impacts the extent to which the voices and experiences of people living with dementia are authentically heard and felt throughout the research. In this reflective article, we come together as three researchers who have used a range of creative and sensory approaches to understand the everyday lived experiences of people living with dementia in different contexts. We present examples taken from three doctoral research studies in which we navigated the creative research space to ensure the voices of people living with dementia are placed centrally and actively within the research process. We pay particular attention to the creative reflexive processes used in each example and explore what ethical research practices look and feel like in the context of creative research with people living with dementia. Our critical reflections lead us to discuss the opportunities that embracing creative approaches may afford in future research with people living with dementia.

## Background

There is growing recognition of the role and value of qualitative, creative research methods in our understandings of what it means to live with a diagnosis of dementia (Keady et al., 2017). It has been acknowledged that traditional qualitative methods (such as interviews and focus groups) are a ‘misfit’ for understanding the complexity of dementia due to their reliance on verbal means of expression alongside an omission of the situatedness of experiences within place and time (Webb et al., 2020). While creative research methods have been proposed as a way to understand the nuance of living with dementia in everyday life, they are often used to represent findings or ‘evoke meanings’ (Leavy, 2018: 10) at the end of a project rather than being seen as a longitudinal, methodologically rigorous, relational journey between researcher and person with dementia across time. This paper is an attempt to bring together what we have learned through our individual research processes, including the ways in which creative research methods can help understand the nuances and complexities of everyday life for people with dementia.

To begin, we define our conceptualizations of ‘creative research’ and ‘complexity’ within the context of dementia. First, we turn to ‘creative research’. Defining what is meant by ‘creative research’ can be elusive, with all approaches to, or adaptations of, traditional research methods having the potential to be labelled as ‘creative’. Regardless of the approach taken, or methods used, Helen Kara (2020) notes that creative research must be underpinned by meticulous research practices, governed by ethics and theory, and have clear implications for practice. It is widely acknowledged that conceptualisations of creative methods fall under five methodological approaches: (1) arts-based research; (2) embodied research; (3) research using technology; (4) multi-modal research; and (5) transformative research frameworks (including participatory, community-based methodologies) (Kara, 2020). While we are beginning to see the emergence and application of creative research practices within the field of dementia (see, for example: Kontos et al., 2021; Moss & O’Neill, 2019; Tsekleves et al., 2020) there has been little reflection to date on what it means to ‘do’ creative research with people living with dementia and how to navigate (and embrace) the complexities that accompany such an undertaking.

‘Complexity’ is a term often associated with dementia, especially in the milieu of health and social climates, systems, and contexts (time, place, space), which interact with the myriad of conditions that fall beneath the umbrella of ‘dementia’. In the context of health research, complexity (or complex intervention) has been approached through frameworks published by groups such as the Medical Research Council (Skivington et al., 2021). Frameworks like this position complexity as mechanistic – a series of cogs and gears that work together to lead to the outcomes we observe (understood through Randomised Controlled Trials or logic modelling, for example) (Gear et al., 2022). These frameworks often use quantitative or mixed-method approaches to understanding, even though they present opportunities to understand and capture some of the nuances of experience that may be underexplored in the context of biomedically-framed complexity frameworks. Our use of the term ‘complexity’ comes from our reading of Gear et al.’s (2018, 2022) work on complexity-theory driven approaches to methodology – approaching our understandings of the lived experience of dementia through transdisciplinary, creative research approaches that allow for the exploration of the everyday (Clark et al., 2020; Kindell et al., 2017), the momentary (Dowlen et al., 2021; Keady et al., 2022), and the embodied and sensory (Campbell et al., 2015; Fleetwood-Smith et al., 2021; Kontos & Martin, 2013).

In this paper, we outline our experiences, contextualised within our doctoral research, in which we used creative research approaches to understand the everyday lived experiences of people with dementia. The findings of these three studies are not the focus of this paper but are presented elsewhere (Campbell, 2019; Dowlen et al., 2021; Fleetwood-Smith et al., 2021). Rather, this paper brings together our collective reflections on what it means to embrace complexity when using creative methods in the context of understanding and foregrounding the lived and embodied experiences of people living with dementia. We note here that our approaches draw parallels with participatory research frameworks, due to the ways in which they challenge traditional asymmetric distributions of power to centralise the lived experiences of participants (Cornwall & Jewkes, 1995). While we do not claim to have all the answers of how to successfully navigate complexity in this area, in this paper we adopt a reflexive approach to understanding complexity when carrying out research with people with dementia. We use examples taken from our individual doctoral studies to contextualise our use of creative approaches as a journey – one that is malleable, relational, and improvisatory (Fleetwood-Smith et al., 2021). Our personal reflexive narratives are embedded throughout the paper alongside the stories of people living with dementia.

## Our Doctoral Studies

Before engaging with the synopses of our respective doctoral studies, please see Table 1: Situating the researchers. Table 1 shares details about our backgrounds and our routes into carrying out research with people with dementia. We share these details to situate ourselves, our biases, and to acknowledge the privileges that our respective backgrounds afford us in approaching and carrying out our work.

The following summaries of our individual doctoral studies provide context for the reflections that we share in this paper.

SC’s (Campbell, 2019) ethnography explored the everyday lives of men living with dementia in care settings. The study followed the daily routines of seven men living in both residential care and in a National Health Service (NHS) dementia assessment unit. Although the study did use some video observation, the main approaches are best described as more traditional ethnographic methods of participant observation (O’Reilly, 2012) and this involved taking of copious fieldnotes, paying attention to the sensory environments and bodily experiences of the men involved. The work is guided by methodologies that purposefully engage with an attunement and perceptiveness to the world around them and the relationship between the senses and the environment (see Abram, 2012; Ingold, 2015, 1987). Ethical approval for this study was granted by an approved NHS Research Ethics Committee with knowledge of the Mental Capacity Act (Department of Health, 2005) [Ref: 11/WA/0147].

RD’s (Dowlen, 2019) research sought to develop an understanding of the ‘in the moment’ experiences of people living with dementia taking part in improvised music-making programme (Manchester Camerata’s *Music in Mind* programme). RD used video-based observation, participant diaries, and video-elicitation interviews to explore the embodied and sensory musical experiences of people living with dementia in the context of the ‘moment’. These methods were chosen to support people living with dementia to ‘relive’ the moment (Keady et al., 2022) outside the music-making sessions. RD took a sensory perspective on analysis, using Sarah Pink’s (2015) approach to re-encountering the music-making process through the senses. Ethical approval for this study was granted by an NHS Research Ethics Committee with knowledge of the Mental Capacity Act (Department of Health, 2005) [Ref: 16/IEC08/0049].

RFS’ (Fleetwood-Smith, 2020) research explored clothing in the everyday lives of people with dementia in a care home, and involved working with people with dementia, care home staff and creative practitioners. The research involved sensory, embodied, and creative methods using multisensory encounters (Pink, 2012) and object handling sessions. The approaches used were developed within the context of the research and were considered co-created as RFS and participants worked together. The approaches used foregrounded knowing ‘through the body’ enabling participation in the research in different forms. Recording methods reflected this focus, with video, audio recordings and fieldnotes used to explore and re-visit research encounters. This work intersects with arts-based research; embodied research; and transformative research frameworks (Kara, 2020) due to the creative practice involved, the embodied focus, and the participatory way in which the research was carried out. Ethical approval for this work was granted by an NHS Research Ethics Committee [Ref: 18/LO/1707].

## Our Approach

As researchers, our paths crossed during our doctoral studies. SC and RD were conducting their doctoral research within the Dementia and Ageing Research Team at the University of Manchester, UK. RD and RFS met at a dementia and arts early career researcher day hosted by the Created Out of Mind team (a transdisciplinary research project focused on understanding the value of the arts for people with dementia) who were resident at the Wellcome Hub (London, UK) [2016–2018].

**Figure 1:**
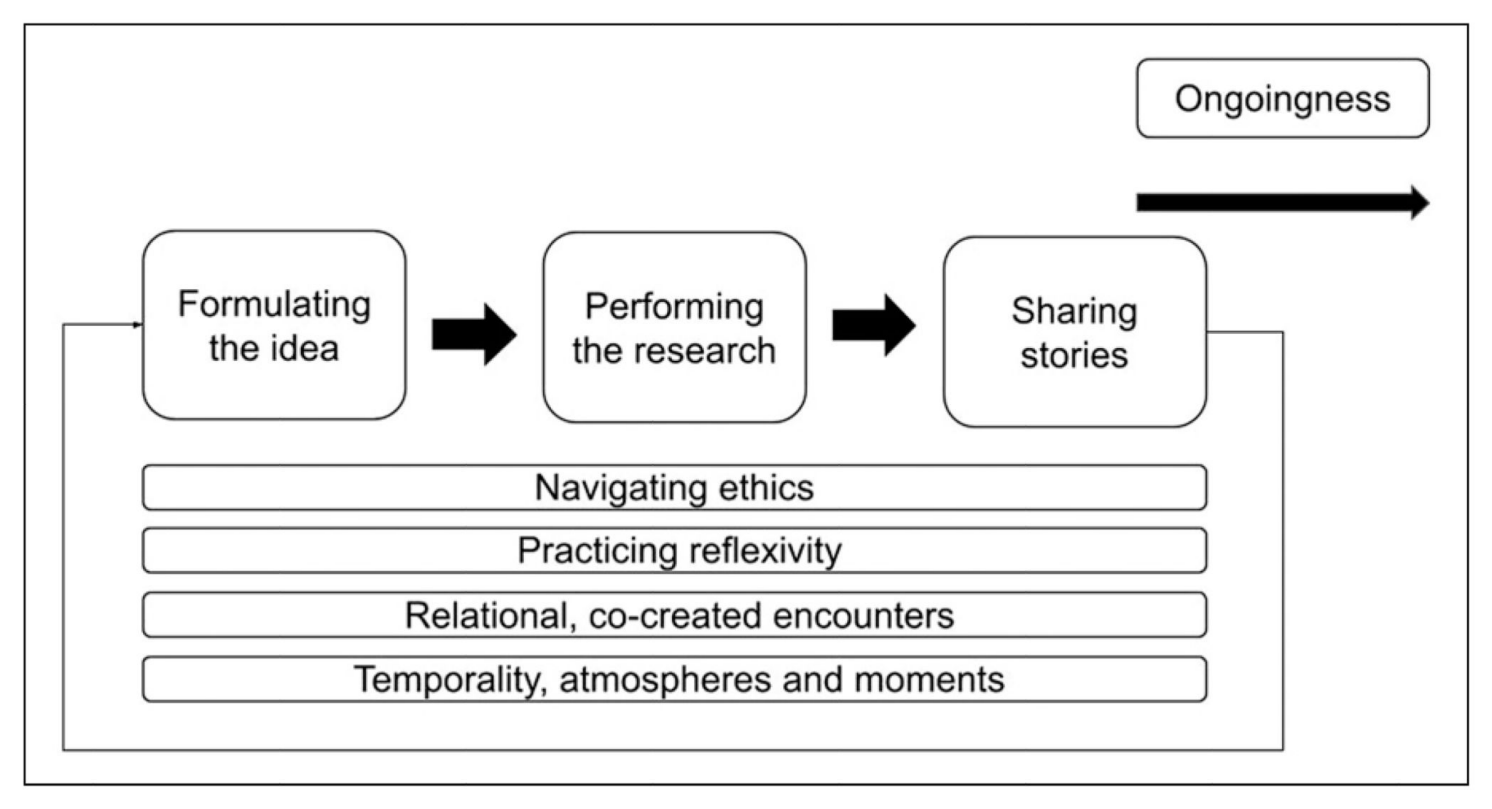
Mapping the interlinked phases of creative research practices.

We came together in early 2021, after our doctoral research, as a community of interest to explore our approaches and understanding of creative approaches and research methods in the context of dementia. While the subject matter of our doctoral work and the methods we employed varied considerably, we understood that by coming together to exchange ideas and reflect collectively on our experiences of using creative methods to navigate and embrace complexity, we could offer learning to the wider community using creative research methods in the context of dementia (Brown, 2019). We came together over a series of virtual reflective sessions to discuss our projects and the intricacies of these practices within dementia research. In August 2022, we took part in an in-person writing retreat where we were able to expand our thinking and map our personal experiences to form the framework for this paper. This led to the development of Figure 1, showing the interlinked phases of (1) formulating the idea; (2) performing the research; (3) sharing stories; and (4) ongoingness.

All these phases of creative research with people with dementia are underpinned by the navigating of research ethics; embedding reflexivity; framing research encounters as relational and co-created; as well as considering the wider temporality, atmospheres and moments that contextualise the encounters. We propose that approaching creative methods in the context of dementia requires the active consideration of these components across the research process and affords the opportunity to understand and embrace the life stories of people living with dementia. There is a significant body of literature surrounding the formulation or co-creating of research ideas with people with dementia in the literature (e.g., Novek & Wilkinson, 2019; Open Doors et al., 2019; Williams & Keady, 2021). For the purposes of this paper, we focus on the later elements in the diagram (performing the research, sharing stories, and ‘ongoingness’). The following section brings together our personal and collective experiences of ‘doing’ creative research with people with dementia.

## Performing the Research

This section explores what it means to ‘do’ creative research through the ethical processes, undertaking fieldwork and analysis, shaping our research encounters, building relationships and the challenges and tensions that can occur. It is important to note that our studies were carried out in England, and this shapes the context in which we worked including (for instance, the ethical processes that we followed).

### Obtaining Ethical Approvals

While the ethical considerations aligned with creative research methods were present across the duration of our individual research processes, we dedicate some time here to discussing ethical approval as an antecedent to being able to ‘do’ the research. While there are more general ethical standards required when using creative research methods (Kara, 2020), there are ethical issues specific to the involvement of people living with dementia in research. The Dementia Engagement and Empowerment Project (DEEP) network (a UK network of groups led by, or actively involving, people with dementia) proposes six principles underpinning ethical research with people living with dementia (DEEP, 2020): (1) working in real partnership; (2) respect and acknowledgement; (3) safety and wellbeing; (4) informed consent and capacity; (5) confidentiality and anonymity; and (6) information that is simple, accessible, and open. While researchers are required to undertake training around, for example, working with adults considered ‘vulnerable’ by the Mental Health Capacity Act 2005, and informed consent processes, such training does not necessarily address the more nuanced ethical implications regarding the design of the research or what ethical practices look and feel like in practice (Fletcher et al., 2019; Puurveen et al., 2015).

Historically people with dementia have been excluded from research, arguably exacerbated by the rigid ethical structures surrounding the involvement of people with dementia in research (Fletcher, 2021). While there is a range of examples of creative, co-produced research with people living with dementia (e.g., Bartlett, 2012; Branco et al., 2017; Capstick, 2012), seeking ethical approval to do this type of research work remains challenging. The ethical approval process when working with people with dementia is a sensitive and contentious area, yet arguably the issues around these processes detrimentally impact research in this area. Research with people with dementia typically requires NHS/Social Care Research Ethics Committee (SCREC) approval, which involves completing an application through the Integrated Research Application System (IRAS). Although there is not space to discuss the approval processes in depth here, these applications involve providing a research protocol and all paperwork associated with undertaking the study. Positivism underpins this framework and the application process rests on the premise that all research is replicable. This does not align with our ways of working, as we consider our research to be of the space, place, and context in which it is undertaken (Webb et al., 2020) and therefore for research ethics to be situated and contextual (Ward & Campbell, 2013).

### Creativity and the Ethics Application Process

Creative research methods can be particularly challenging to position when compiling documents for the ethics application process. The very act of writing our respective applications was therefore creative. For example, RFS built flexibility into her protocol so that she was able to respond to the research as it unfolded, yet this generated a large body of supporting paperwork to justify this approach, the sheer volume of which was negatively commented on at ethical review. Similarly, RD’s application was supported by 35 supporting documents, including distress and safeguarding protocols. She created information booklets that used images to guide people living with dementia through the research process but was told to use these only as a ‘social nicety’ rather than a way to keep people with dementia informed about the research.

While we developed rigorous ethical protocols to perform ‘ethical research’, we each factored flexibility into our approaches to ensure people with and without capacity to consent were represented within the research. For example, RD developed a distress protocol in line with recommendations from the ethics board, in which the person with dementia would be removed from the research (and as a result, the music-making group) at any sign of distress. In reality, the music-making process resulted in many emotional experiences (i.e., crying) being shared by group members that could have been interpreted as ‘distress’. This meant that the distress protocol required acknowledgement of the musical process and the research ethics surrounding the research, but also a recognition of each nuanced moment and its relationship to the music-making activity as well as the research methods (i.e., video observation). SC’s ethics application was embedded within the application for the colloquially known Hair and Care project (RES-061–25–0484) and this application initially received an unfavourable response due to concerns of involving people living with dementia who did not have capacity and around the use of video methods, despite the Principal Investigator and SC submitting reams of documentation to support the application process. It is also important to note here that video methods are not new in social research (Pain, 2012); that the Principal Investigator had used video methods previously; and that both SC and the Principal Investigator undertook training in the use of video methods in research. Additional resources submitted included an ethical protocol that sought to outline consideration of all potential contextual ethical concerns that could occur in the field and ways to respond, importantly including ways to support a participant’s wellbeing during the study (see Ward & Campbell, 2013). This leads us and others to question whether all research ethics committees have the relevant knowledge, expertise and understanding regarding the use of creative approaches within qualitative social research and those that include people who do not have capacity to consent (Fletcher, 2021; Hammersley & Traianou, 2012; Ward & Campbell, 2013).

### Gaining Ethical Knowledge

Although these ethical processes have been challenging and time-consuming, it does mean that as creative qualitative researchers, we have become highly skilled and reflective in ethical research practices. We have undergone rigorous review processes and have further reflected on research ethics in practice, considering the impact of our relationships and the ‘in the moment’ ethical challenges faced in undertaking creative qualitative research (Hodge et al., 2020; Novek & Wilkinson, 2019). We have focused on the wellbeing, engagement, empowerment of people living with dementia both with and without capacity and we have been active in ensuring that their experiences are heard, seen, or felt. These ethical undertakings also enable us to connect with other researchers doing similar research and to build a community of supportive peers, where these ethical dilemmas can be further considered and reflected upon. Thus, it is important that the ethical processes for undertaking creative research become more attuned to these kinds of relational dialogues if we are to ensure the voices of people living with dementia are included in research.

### Creative Research Encounters

After obtaining the required ethical approvals, each of us entered the field to recruit and subsequently work with people with dementia using creative methods. In this section we consider how we ‘do’ creative research and present reflections and examples from our three unique research studies and encounters. We explore ways of being engaged creatively with research, not simply using creative methods but also through being actively engaged as ‘creative’ researchers through practices of attunement and attentiveness. As researchers working alongside people living with dementia it is important to find ways of working that support and enable people living with dementia to participate in research studies. All studies by Campbell (2019), Dowlen et al. (2021) and Fleetwood-Smith et al. (2021) worked alongside participants living with more advanced dementias and who were not able to give informed consent themselves. These studies sought methods to ensure the participants’ ‘voices’ would be heard and included in the research, and hence all three studies required consideration of methods that could capture experiences other than verbal exchanges.

### Attunement and Attentiveness as Creative Research Qualities

Creative approaches in the context of these doctoral studies consider concepts of attunement, attentiveness, and perceptiveness relating to the nature of everyday experiences. Pink (2012) notes that, for researchers to access and engage with the senses of others, they must first be attuned to their own sensory perceptions. We agree and suggest that this kind of ‘full-bodied’ approach to doing creative research is important (Ward & Campbell, 2013b). Arguably, creativity in research enables a more interconnected and multi-dimensional engagement with the experience of others from one’s own body. Indeed, Kara, in her 2020 edition of *Creative Research Methods: A Practical Guide* (Kara, 2020), now includes embodied research within her conceptualisation of what fits into a definition of creative methods (which did not appear in the 2015 version). We propose that sensory methods that complement the embodied should be drawn further into our understandings and use of creative research methods, especially within the context of dementia.

Les Back’s (2007) book *The Art of Listenin*g notes that there is an ‘artfulness’ (p. 8) to research that requires attentiveness through the use of the whole body or through our senses to engage with participants and data in the field. This ‘artfulness’ speaks to a sense of creative endeavour. These qualities of being attuned (Stewart, 2007) or attentive (Back, 2007) enable and support a way of being in the world as a researcher. Back (2007) suggests that sociological listening ‘involves artfulness precisely because it isn’t self-evident but a form of openness to others that needs to be crafted, a listening of the background and the half muted.’ (p. 8). The idea that this kind of engagement with research participants and their lives is itself an artful and crafted practice has guided the work reflected on within this paper.

### Embodied and Sensory Ways of Knowing

RFS’ thesis provides an example of an attentive knowing through her body when with participants:

P1 (PWD) talked of being prevented from being naked on a hot day:

“You [referring to herself] are as close to naked as they will let you get – it is just

My T-shirt and my skin!” She pulled her top away from her body and looked

Down her front, seemingly checking whether or not she was wearing a bra.

The room we were using was stiflingly warm. The vinyl chairs exacerbated the heat, and the air was thick and heavy – the research encounter was curtailed because of this.

In this extract from RFS’ (2020) sensory ethnography, she acknowledges the heat experienced by the participant, the hot day that, in the end, drew the session to a close sooner than planned. The feeling of the stifling air was captured in these notes, and actions undertaken by the participant. Even in this short extract, the sense of warmth is experienced, through the sensation of heat on vinyl, and the thickness of the warm, heavy air.

In SC’s (2019) ethnographic observations, she too is attuned to what she describes as an atmosphere of waiting. She writes in her fieldnotes:

I move to sit in the dining area where Don and another man are sat on different round tables to each other…the other man is whistling and tapping his wallet against the table…Don begins to sing and [then] it drifts off. Don sits watching the nurse’s office where there is some activity, nurses are coming and going. It is like we are all waiting. (2.40p.m. 23^rd^ February 2012)

The experiences in this extract reveal full-bodied attentiveness and attunement to the bodies of others and to the sensescapes, which are felt through the bodies of both researcher and participants. The idea shared in this extract is that they are all waiting for something to happen, as they sit, researcher and participants and other residents, within their own personal spaces, watching, tapping, and waiting whilst the nurses are busy and active. This creates a feeling within the short extract of the atmosphere of the space where the fieldwork took place. As we will go on to discuss, the capturing of these sensorial experiences through writing and words can be limited. However, in these fieldnotes there is a clear sense of how much the researchers’ bodies are engaged in experiencing similar atmospheric or bodily senses to those of the participants.

We have found it important to attend to these full-bodied ways of knowing across the creative research journey. RD for example, noted in her research that the analysis video data led to her own re-living of the research context (Pink, 2015) – experiencing goosebumps during poignant musical moments and laughing as the group laughed together when reviewing video segments. Thus, the importance of these approaches is in understanding that there are ways of knowing that are embodied and sensory (Mason & Davies, 2009) and that this kind of ‘listening’ requires qualities and approaches that are crafted artfully – creatively (Back, 2007).

### Flexibility and Openness in Creative Approaches

Our aim here is to highlight the importance of a flexible and open approach, as research of this nature is not predictable. In the same way that creative practitioners are alert and flexible to inspiration and to the evolution of an idea, creative researchers must inhabit a flexible and open attitude to undertaking research (Leavy, 2020). When researching alongside people living with dementia, there can be unpredictability in terms of how someone might be feeling at any given moment. Hence, researchers need to be prepared to change their plans or amend their focus during data collection. This aligns too with the flexibility and openness required in care work, within which bodies cannot be easily scheduled, and are often unpredictable (Cohen, 2011). For example, it is not always possible to say how long supporting someone to get dressed or bathed in the morning will take, and there are times when someone may not wish to get dressed at that particular time. SC (2019) notes that when she arrived to observe a shaving activity with one participant, she found that they had got up very early and had already been shaved by the night staff; on another occasion with a different participant, they did not want to be shaved at the time scheduled, and certainly did not want to be observed.

RD (2019) also noted how the creative methods used within the research did not suit every research participant. One participant, Mary (not her real name), responded to the re-watching of videos with only “that’s nice” or “yes, I liked that”, but was more open to sharing her experiences in the format of a more traditional semi-structured interview. Creative research, then, requires researchers to be flexible, open, and prepared to adapt their research plans.

### Challenges for Creative Research Endeavours

The three studies considered here for their creative approaches to ‘doing’ research all used videography as a tool for capturing the bodily and sensory experiences of the participants. Pink (2007) notes that video observation work enables the researcher to revisit the sensory spaces that they inhabited when undertaking analysis. Using film for data collection is followed by an in-depth layered approach to analysis and can lead to a deeply detailed analysis of even a very small amount of film. All three studies were flexible and open in the field, taking time to build relationships with participants and to acknowledge when research sessions were not working, needed changing, or needed to stop altogether. What emerges, then, from using creative methods and embracing creative approaches, are the challenges of time, and resource. For researchers to be truly attentive and attuned takes time: time to obtain ethical approvals; time in the field; time in analysis; and time sharing stories. This is challenging for research studies limited by funding periods, and hence consideration has to be given to what is achievable in particular timeframes, particularly if researchers wish to be flexible and open, and to use methods that can generate large amounts of data for analysis.

This section brings together what it meant for us to ‘do’ creative research with people with dementia. We explore processes ranging from the creative ways in which we approached the ethical approval process to our analyses of our research. Personal reflections and extracts from our work with people with dementia illustrate this work ‘in practice’. The following section explores how we approach disseminating creative qualitative research and the opportunities and challenges this can involve.

## Sharing Stories

Presenting creative research is complex and involves carefully considering meaningful ways in which to communicate and express our work. Examining what we share, how we share it, and who we share our research with, is not simply a question of the approaches that we use, but, as Pickering and Kara (2017:300) note, this is a “deeply ethical arena”. As we have discussed, ‘doing’ creative research involves being responsive, open, flexible, and attentive yet extending these practices to the sharing of participant’s stories is highly complex when navigating traditional ethical and academic frameworks. For example, when presenting a conference paper and speaking the words of a participant, we choose what we read, and their words are defined by our intonation, pitch, pace, and tone; thus, in some sense, we ‘perform’ these words (Pickering & Kara, 2017). In this section, we critique the extent to which such practices shape what we present, discuss, and share, and the extent to which audiences engage with our research.

### Sharing Words

Traditional formats often limit how we communicate our research and do not convey the tenets underpinning the work. Each of our studies were contained within a traditional thesis format. Yet, our research involved learning through our bodies and attending to the specific time, place, and context in which our research was carried out. Reducing these learnings to a series of written findings often negates the embodied aspect of the research (Kriger, 2019). As Pickering and Kara (2017:307) note, we must “identify and acknowledge the nature of our sacrifice” when we consider how we present and share our work.

Those working with creative research methods advocate alternative ways to present and disseminate research, claiming that traditional formats can not only limit how accessible research is, but can also limit the very scope of the research itself (Kara, 2020; Mannay, 2015; Pickering & Kara, 2017). For example, traditional written forms of dissemination, although highly in-depth, often fail to express and communicate the complexity of embodied and sensory-focused research. Further, the need to ‘fix’ words on a page can limit how we engage and respond to research, negating the ways in which such research is often ongoing, unfixed, and changeable.

### Sharing Images

**Figure 2:**
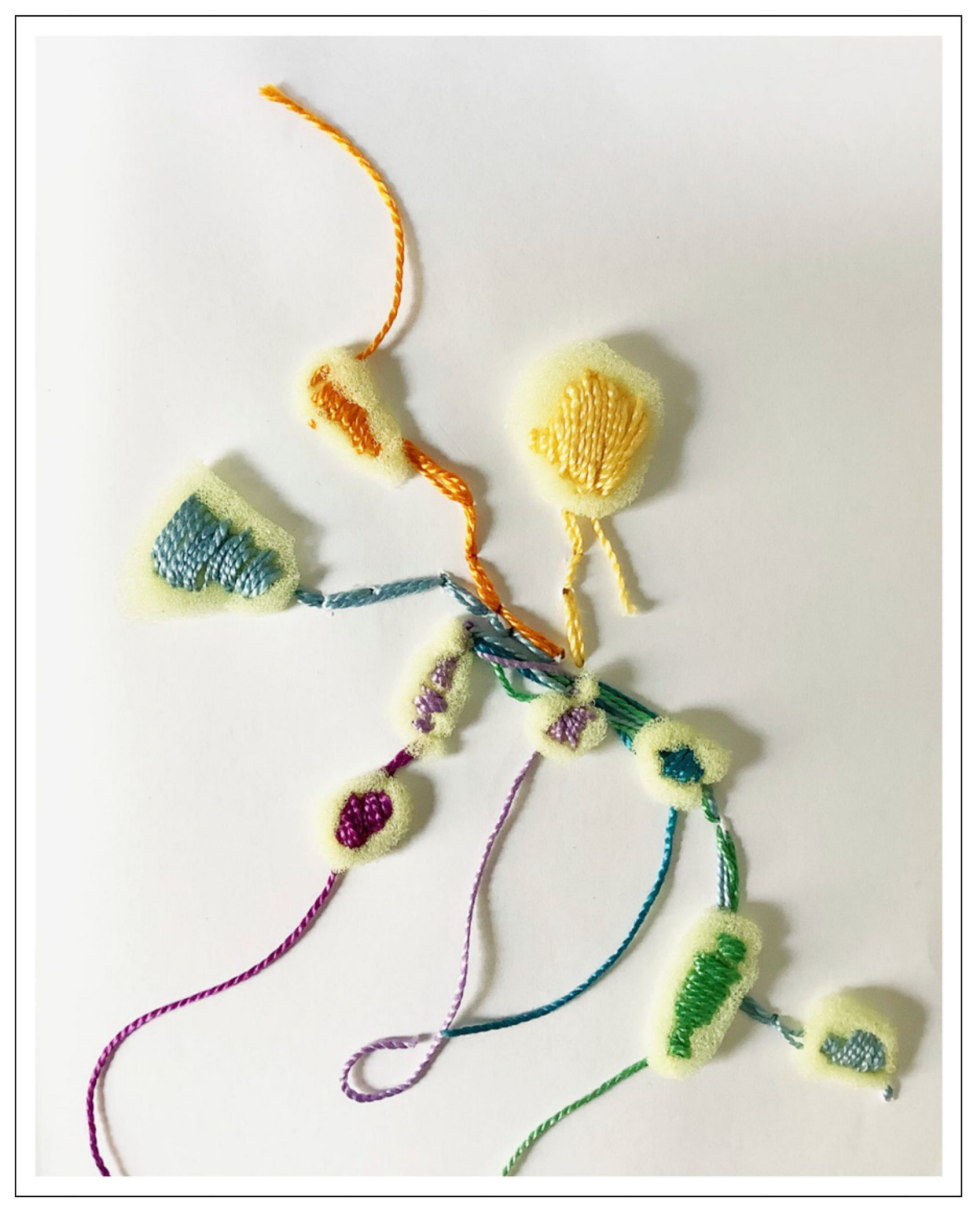
RFS’ reflexive textile mark-making.

In an attempt to express and convey a sense of the embodied encounters that take place within research, researchers often use images to add richness and depth to their writing and support the ways in which they think through their work. For example, RFS used creative practice to engage reflexively with her research and explore concepts that were difficult to articulate through words alone. She then used images of her practice (see, for example, Figure 2: Reflexive textile mark-making) within the thesis to illuminate her thinking.

**Figure 3:**
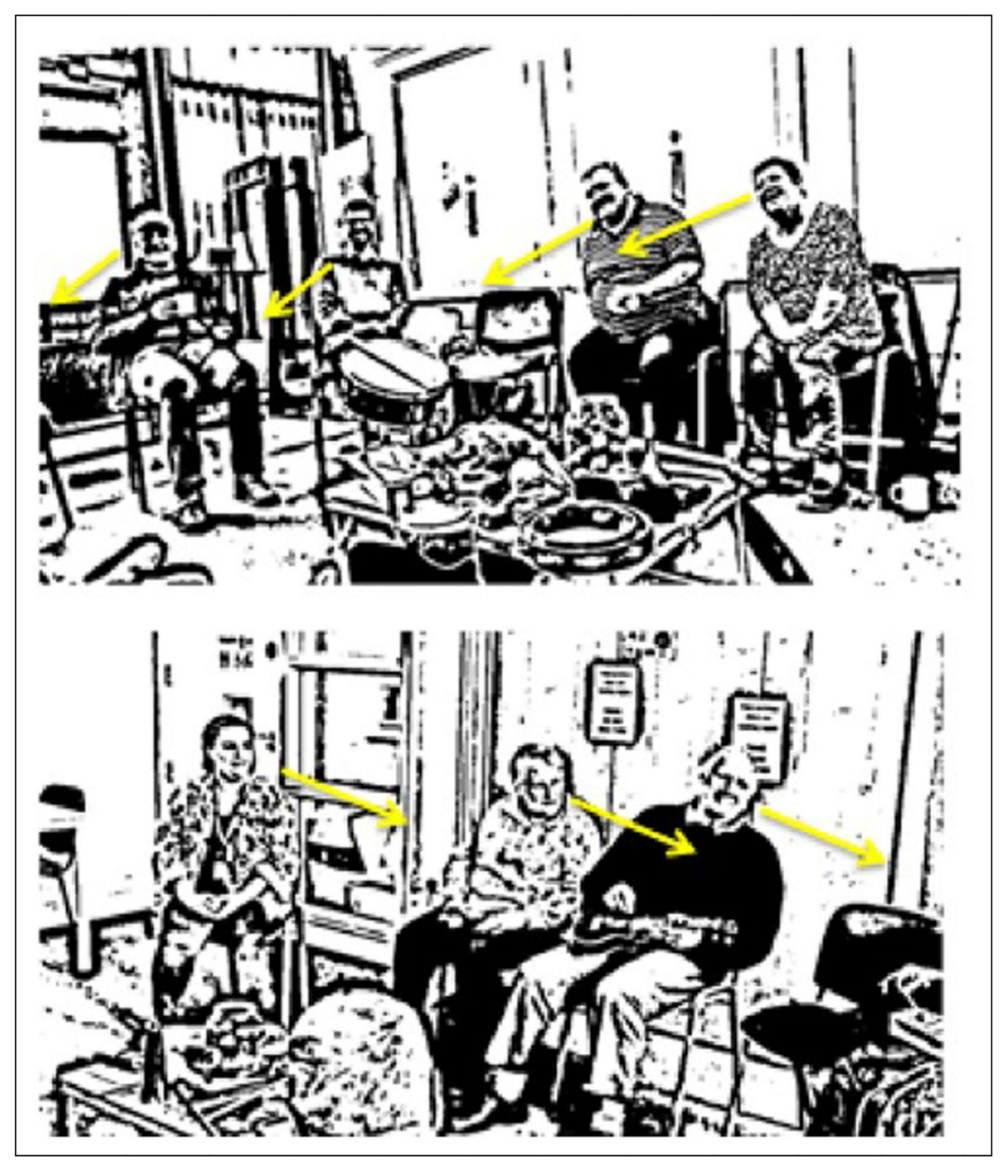
Arrows added to RD’s images to convey group gaze.

RD took video stills from the music-making sessions with people living with dementia to support the narratives of the ‘in the moment’ experiences she had encountered whilst part of the group. Following ethical guidelines on the presentation of images of people collected through video-based methods (and on the recommendation of the ethics committee), images were altered to protect the identities of those involved. However, this resulted in the potential de-humanization of the research participants, distorting the images beyond recognition of a body existing as a material object in time and space. This led to the need for additional clarification through words. For example, additional notation often had to be added to images, to clarify who was living with dementia (something that was never discussed in the group itself), with arrows indicating the direction of gaze to illustrate concepts such as ‘musical spotlighting’ (see Dowlen et al., 2021). Figure 3 presents one such example of an image where additional notation was required to present the findings in a traditional way. RD reflected in her thesis how sending these distorted images to participants to ensure they were happy for their use seemed meaningless, given their identities had been removed from the images.

**Figure 4:**
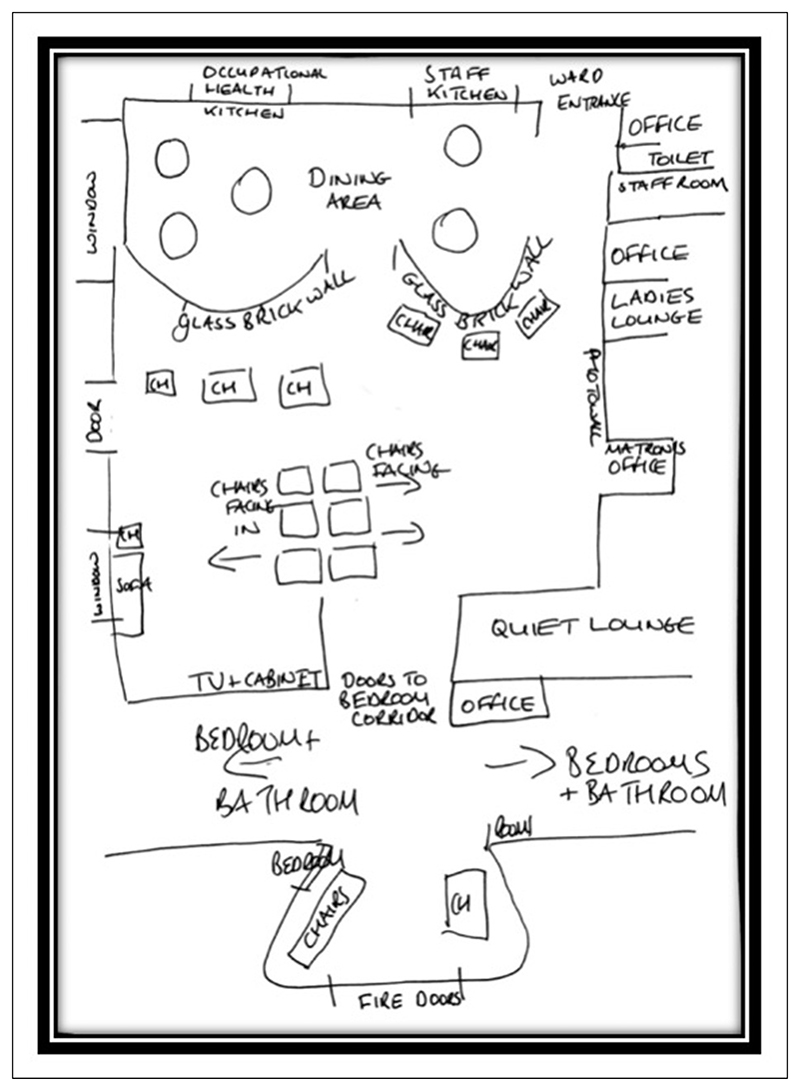
SC’s floor plan of a communal care home space.

Disseminating images therefore involves ethical concerns around what we ‘reveal or conceal’ (Kara, 2020), especially when sharing images in which a person or place could be identified. For example, as discussed, participant anonymity typically makes it necessary to distort an image, leading us to question the extent to which such images are actually useful when sharing findings. This is especially sensitive when working with people with dementia, as the condition is highly stigmatized and distorting someone’s face may be linked with rhetoric around loss of selfhood and thus exacerbate stigma associated with the condition. This also impacts how we share the spaces in which we are working. For example, within SC’s work she used diagrams of the communal spaces of the care settings in which observations took place to try and create a visual understanding of the spaces where the men in her study were living out their everyday lives (see Figure 4). The diagrams aimed to provide a floorplan of how material objects were positioned (for example, seating, televisions, and decorations), which were then discussed within the text. Yet diagrams such as these are devoid of the people, materials, and sensations of place, so still limited in what they can convey. Therefore, as Kara (2020) notes, it is essential that the use of creative approaches is meaningful and appropriate for both the research and the audiences we wish to engage.

### Sharing Through Creative Practice

We often see creative practices, such as poetry, drama, and visual arts, cited as useful tools with which to communicate research findings. This is a growing methodological practice and can be considered a useful way to broaden the reach of academic work and disseminate findings to the public (Leavy, 2018). Such approaches can be more accessible than traditional academic formats and are often used to support action around social issues (Boydell et al., 2012; Leavy, 2018). For instance, Tierney et al. (2021) found that creative approaches supported patient and public involvement work with people often under-represented within research and led to the identification of research priorities. However, Bartlett (2015) notes that such work necessitates critical engagement around how the arts are used and why, and the impact that they may have on the research. For example, Bartlett found that the very nature of the arts practice drew audiences’ attention to the aesthetics of the artwork, as opposed to the findings underpinning the work. While this was seen as a limitation within Bartlett’s work (2015), such engagement with aesthetics was an important aspect of RFS’ research. Within RFS’ (Fleetwood-Smith et al., 2021) study, she worked with creative practitioners to explore how findings could be explored and reconsidered as a series of objects, materials, and images. Within this translation of findings, she sought to promote conversations around taste and aesthetics. The objects, materials, and images were used not as representations of research findings but as ways to facilitate different types of engagement with the project (Fleetwood-Smith et al., 2021). This was challenging to navigate, and attempts to find precedents of such practice drew upon design-led approaches such as prototyping workshops (see e.g., Lab4Living, n. d.).

### Inviting People ‘into’ our Research

The use of objects, materials, and images in our respective projects speaks to the ways in which researchers are increasingly challenging how we present and discuss creative qualitative research. These approaches involve inviting people ‘into’ the stories of such work. For instance, drawing upon non-representational theory and more-than-human approaches to research (e.g., Andrews & Grenier, 2018; Barron, 2021), Manchester and Willatt (2022) used poetry, vignettes, and images to share stories from their work with older adult co-researchers. Their paper, presented at the British Gerontology Society’s Annual Conference, sought to allow their data to ‘dance a little’ and involved multiple ways in which to engage the audience. This spoke to the ‘liveliness’ of their data and created room for contemplation and reflection. Presenting research in this way is a significant shift away from the notion of static, fixed findings and embraces how research ‘data’ is multi-layered, contextual, and co-created.

This openness to presenting findings connects with practices, for instance, from design research that can involve the use of exhibitions, workshops, and public engagement events as ways to share, explore and co-create findings with different audiences (e.g., Lab4Living, n. d., online). Exhibiting can be a powerful way of inviting people ‘into’ research, as artworks can challenge audiences to think differently about topics. Depending on the curation and pieces, exhibitions can ‘speak’ to all the senses (Lupton & Lipps, 2018) and allow for accessible engagement through the different forms works may take. For example, the ‘Sensing Spaces of Healthcare’ (Sensing Spaces of Healthcare, 2023) project, on which RFS is creative researcher, is commissioning artworks that will be exhibited at each NHS study site. The commissions will allow visitors to engage with themes underpinning the project. Artworks will be multisensory and therefore support engagement in the project in multiple ways. We have also found the Economic and Social Research Council’s Festival of Social Science to be a helpful forum in conveying the findings from our research to members of the public.

The nature of approaches, such as events and exhibitions, speaks to increased attention around flexibility, openness, and participatory ways of carrying out research, which involve embracing subjectivity and the intricacies of the research process. Such qualities are arguably essential when communicating our research findings. Yet, challenging how we present and discuss research is complex. As Boydell et al. (2012) claim, this involves questioning our beliefs around knowledge production and what it is we seek to communicate. Such approaches are ‘risky’ and difficult to adopt due to traditional academic frameworks and funders’ requirements. Taking such risks can be particularly challenging for those early in their careers due to, for example, job precarity and power relations within academia.

This section has explored the opportunities afforded when sharing research stories in creative ways. Creative approaches do not provide an exhaustive way to interpret or represent the complex knowledge generated within creative qualitative research, yet they can engage audiences in new ways and enable alternative ways of knowing and thinking. Employing multiple methods to convey and share the stories of this work is essential in foregrounding the complexities of this research and is an essential part of ethical practice (Pickering & Kara, 2017). Embracing a menu of ways in which to disseminate our research can support the ongoing nature of this work by promoting continued engagement with the stories shared. We build upon the notion of ‘ongoingness’ in the following section, which considers how we bring research projects to a close.

## Ongoingness

The complexity surrounding the unfixed nature of this work is important to consider, not only in terms of how we share our research, but how we bring projects to an end. For example, Fletcher et al. (2019) advocate ‘hanging out periods’ with potential participants prior to starting research, yet little attention is given to how projects end, and how this impacts participants and researchers. We viewed our participants with dementia as invaluable contributors and carried out research *with* them, as opposed to carrying out research ‘about them’ (Phillipson & Hammond, 2018). We worked with our participants over considerable periods of time and got to know them well. For example, RD entered the music-making groups as a participant-researcher, taking part in the music sessions with the group as well as visiting people in their homes. RD still remembers how each person within the group liked their tea/coffee even though she has not had contact with them since the end of the project in 2017. Project endings can feel artificial, as they ‘end’ in accordance with funding timelines and ethical requirements and this does not account for how this research is carried out in practice. For example, we are no longer in touch with those who participated in and co-created our research, yet we continue to think about them as we share our work (Clark & Campbell, 2023).

Working with methods that foreground attentiveness, flexibility, and collaborative practices shapes our work in the ‘field’ but should also inform how we bring such work to a close. The disentanglement from the networks and relationships forged during such projects must be sensitively navigated. We also question how far this disentanglement is necessary and whether we could re-think the tensions and constraints around these imposed endings. Exploring complexity in this work involves acknowledging and illuminating the ongoing nature of these practices. Our reflections illustrate that greater attention and resource should be paid to ensure that ethical practice underpins how we bring research projects to an end. Reflecting on the notion of ongoingness leads us into our concluding discussion, in which we summarise what creative methods offer researchers working with people with dementia.

## Final Reflections and Conclusions

Using creative research methods when working with people living with dementia provides vast opportunities for understanding people’s lives. Many of the creative methods discussed within this paper involve working with people, embracing the ways in which we work with people living with dementia ‘in the moment,’ co-creating our understandings. These ways of working involve attending to embodied and sensory ways of knowing, alongside being flexible and attuned to the experiences of those taking part in the research. We have situated our understandings of creative research methods in the framing of ‘complexity’ and how the experience of dementia is made up of many systems and inter-related processes that mean it is difficult to predict what may occur (Cohen, 2011; Gear et al., 2022). But we must also recognise the ways in which people with dementia situate themselves and their position as citizens in the world. This is often not through verbal exchanges but through their embodied, relational experiences with the world (Kontos et al., 2017). Here, creative methods offer a unique approach to understanding complexity in the context of the lives of people with dementia, and allow us as researchers to hear, feel, and sense their experiences.

One thing we have touched on, but not explicitly discussed within the body of this paper, is whether it takes a particular type of researcher to do this kind of creative, embodied, and sensory work with people with dementia. Indeed, Phillipson and Hammond (2018) in their review of innovative approaches to creative research note:

Arguably, the majority of these studies are theoretically informed, creative, attentive, reflexive, intentional, careful and pragmatic. This implies that they have been conducted by researchers who view participants with dementia as valued informants and who have taken time to get to know their participants, often designing research for them and with them, rather than just about them. (p. 10)

While we are by no means claiming to tick the boxes for the criteria of such researchers, we have engaged with these methods in a way that has been driven by our own life experiences – for example, as children or grandchildren of people living with dementia; as advocates for the use of creativity and the arts as means of self-expression; and the personal value we place on creativity in supporting our own wellbeing. We must also acknowledge that we are all white women, and we have privileges to take risks within our research that other people may not have. This also limits our perspectives when interpreting what we hear, see, and feel through the research process.

We are committed to developing our approaches to research further as we continue to work with people living with dementia and we embrace the ways in which our practices are ongoing, unfixed, and ever-developing.

## Conclusion

Overall, our experiences of using creative research approaches to understand the everyday lived experiences of people with dementia have shaped us as researchers working in this field. We have taken what we have learned through our doctoral studies and applied it to our postdoctoral research, including supervising research students taking the leap into creative methods. We also acknowledge that our learning is ongoing, and very much shaped by the people with dementia who we interact with through our research encounters as well as the contexts (place, space, time) in which we work.

While the use of creative methods is complex, time- and resource-heavy, as well as challenging at times, we hope that more researchers see the opportunities these methods afford in centralising people with dementia in the research that we do. The ways in which research can be approached in this area are wide and varied, but with an ongoing dialogue of research processes we may begin to find methods that have a greater ‘fit’ in understanding the lives of people with dementia and supporting them shape the research agenda, process, and outputs (Webb et al., 2020).

## Figures and Tables

**Table 1:** Situating the Researchers.

**Robyn Dowlen** Demographic information: White british, cisgender, non-disabled, middle-class woman. Child of first-generation parents with university degrees. Disciplinary background: Psychology and music Route into dementia research: I entered the field of dementia after my postgraduate degree in research methods in psychology. I Had not had any teaching on qualitative research methods ahead of my postgraduate studies but was drawn to their role in understanding people’s lived experiences of different health conditions. My interest in dementia in particular stemmed from personal experience of dementia within my family as a teenager and observing the challenges within the transition to care and the impacts that it had on my grandparents, my father, and wider family. My interest in the creative arts stems from the many opportunities I have had to engage with music and theatre across my lifetime – from having violin and singing lessons from age 5, to performing in school musicals and professional choirs in my teenage years.	**Rebecka Fleetwood-Smith** Demographic information: White british, cisgender, non-disabled, middle-class woman. Child of first-generation university graduates. Disciplinary background: Fashion textiles design and applied psychology Route into dementia research: My work with people with dementia grew out of my interest surrounding the impact of creativity and design in people’s lives. I Entered the field after working on an arts and health charity project. While undertaking my PhD a close family member received a diagnosis of dementia, and this continues to drive my work and practice. My background shapes my approach to carrying out research and has led to my interest in designing and developing creative approaches when working with people with dementia. Aside from my research, I regularly volunteer to run creative workshops for (and with) older adults and people with dementia and am passionate about the power of the arts in our everyday lives.	**Sarah Campbell** Demographic information: White British, cisgender woman, non-disabled, working-class origins, first-generation university graduate as a mature student. Disciplinary background: Anthropology; Women’s Studies/Gender Studies; Social Scientist. Route into dementia research: My work within dementia began whilst working in the voluntary sector with people growing older with learning disabilities where I worked on a project which explored dementia and learning disabilities. Around 2007, my father was diagnosed with Dementia, and at a similar time my Father-in-Law was diagnosed with Alzheimer’s Disease, and I developed a personal interest in dementia. A short while later I took a role as a Research Assistant on a short-term contract at the University of Manchester within the Dementia and Ageing Research team. This role led to subsequent opportunities on other projects and I undertook a (part-time) PhD in the field of dementia studies. Over the last 14 years I have been deeply immersed within the field of dementia studies both personally and professionally. Furthermore my background studying anthropology has led me to engage in research that is immersive, and centred around everyday lived experience, this has led me to creative methods – and methods that engage in sensory and bodily engagement. Prior to this my work in the voluntary sector was engaged in participatory methods of working in research and policy. This has led me to want to find ways to ensure the ‘voices’ of people living with dementia are central to my work. I am also deeply passionate about creative arts in everyday life, including my own life.
